# The Bill for the Registration of Midwives, 1897

**Published:** 1897-03-06

**Authors:** 


### THE BILL FOR THE REGISTRATION OF MIDWIVES, 1897.

Mr. R. Humphreys, hon. secretary of the Midwives Bill Committee, writes: Will you permit me to direct attention to one or two misleading remarks which have crept into your able article. May I first point out a typographical error in the title you give the Bill. It is not a midwives' Bill, but a Bill for midwives. It might have been better to have headed the article in the way this letter is headed. May I also remark that the term "Midwifery Nurse" has not been dropped in the present Bill. It has never appeared in any Bill before Parliament, and is generally acknowledged, I believe, to have been inserted with a view to confusing the question at issue. With regard to the definition of the term "Midwife," a Bill has become necessary for the registration of midwives, because a midwife is only limited by traditionary use, and there is no limit in law to the scope of her practice. The term "Natural Labour" has been dropped out of the definition, because one of the chief objects for which the Midwives Board is to be created is to prescribe the conditions under which a midwife should practise, and to punish her if she shall exceed her proper sphere of practice. It is obviously a very dangerous thing to ask a lay body to discuss highly technical questions such as this. As to attempting to restrict the practice of midwifery by unqualified persons, the battle against unqualified practice is being carried on by medical bodies specially constructed for this purpose, and has no connection with a Bill such as the one under discussion. I may say, however, that no Bill which proposed to prevent a woman from employing any person she desired to attend her in labour would, in the present state of opinion in the Houses of Parliament, have the slightest chance of being heard. The limitations of the practice of midwives are to be regulated by the General Medical Council, and since, as you say, "there is no necessity to define a midwife any more than in a muzzling Act there is any necessity to define a dog." There can be no doubt as to what limits will be imposed upon the practice of midwives. The Bill is intended to refer only to women, and, with regard to the powers of the supervising authority, it is obvious that the same person could not well be made the judicial and the administering authority. He will have the administration of the rules made by the Midwives' Board, leaving to it to decide on complaints he brings before it. He will, undoubtedly, be able to suspend her from practice under the rules laid down by the board, as in cases of puerperal fever. (See clause 5, subsection G.)

*** We insert the above letter because we presume that it is to be taken as expressing the opinions of the Midwives' Bill Committee, by whose secretary it is written, but we beg entirely to dissent from the statement that the remarks in our article on the subject were in any way "misleading." It is mere trifling to object to a "Bill for Midwives" being called a "Midwives Bill," or to protest against our saying that the term midwifery nurse had been "dropped," because forsooth it had never been in any Bill before Parliament! Putting on one side, however, such trivialities, we would point out that the letter is a very good instance of the confusion of thought which seems to reign among many people who discuss this subject. Mr. Humphreys says "a Bill has become necessary for the registration of midwives, because a midwife is only limited by traditionary use, and there is no limit in law to the scope of her practice." From this one might imagine that the Bill proposed to impose some limit on the scope of her practice. But such is not the case. It only proposes to impose such limits on those who register; those who do not register are not interfered with in their practice in the slightest degree, they are only prevented from assuming the name! It ought, we think, to be clearly understood that although the great plea on which the framers of this Bill go to Parliament is the danger to which the women of England are exposed in consequence of the practise of midwifery by unqualified persons, there is not a word in the Bill to prevent unqualified persons from practising. Mr. Humphreys himself says that "no Bill which proposed to prevent a woman from employing any person she desired to attend her in labour would, in the present state of opinion in the Houses of Parliament, have the slightest chance of being heard." If this is so why trouble about it? We do not, however, believe that it is so; and we think that if it can be made clear to the public that the danger to the poor women of England is as great as it is represented to be, Parliament is more likely to intervene for their protection than for the passage of a Bill which confessedly does not attempt to prevent the practise of midwifery, even as a means of earning a livelihood, by the most incompetent of women. Parliament has not hesitated to regulate the milk trade, and to place butchers and bakers under inspection, and there is no reason to believe that it would hesitate any more to impose restrictions on the practise of midwifery if only it could once be convinced that women's lives are being sacrificed for want of such restrictions.

